# Knowledge-based model for automated multi-isocenter total marrow and lymphoid irradiation planning across standard and large patient anatomies

**DOI:** 10.1016/j.phro.2025.100781

**Published:** 2025-05-20

**Authors:** Manuela Meraldi, Nicola Lambri, Damiano Dei, Piera Navarria, Giacomo Reggiori, Ciro Franzese, Stefano Tomatis, Cristina Lenardi, Marta Scorsetti, Pietro Mancosu

**Affiliations:** a Radiotherapy and Radiosurgery Department, IRCCS Humanitas Research Hospital, Via Manzoni 56, 20089 Milan, Italy; b Dipartimento di Fisica “Aldo Pontremoli”, Università degli Studi di Milano, 20133 Milan, Italy; c Department of Biomedical Sciences, Humanitas University, Via Rita Levi Montalcini 4, 20072 Milan, Italy; d National Institute for Nuclear Physics (INFN), Milan Division, 20133 Milan, Italy

**Keywords:** Knowledge-based (KB), Total marrow and lymphoid irradiation (TMLI), Radiotherapy (RT), Volumetric modulated arc therapy (VMAT)

## Abstract

**Background and purpose:**

Total marrow and lymphoid irradiation (TMLI) planning is challenging. This study evaluates whether a knowledge-based (KB) model for TMLI delivered using volumetric modulated arc therapy (VMAT) can achieve clinically acceptable dose distributions through fully or semi-automated optimization and whether a single model is effective across varying patient anatomies.

**Materials and methods:**

Fifty-one consecutive VMAT-TMLI patients were selected. A KB model was trained using 30 patients treated with standard configurations (5 body isocenters). Validation included two cohorts: 10 standard patients and 11 patients with a larger anatomy treated using separate isocenters for the arms (4 body and 2 arms isocenters). Two planning approaches were explored: fully automated (AutoKB), and KB with manual adjustments (HybridKB) by a planner with no prior experience in TMLI. KB plans were evaluated against clinical plans (CPs) using paired t-tests.

**Results:**

The KB model reduced mean doses to major organs-at-risk (OARs). For standard configurations, mean OAR doses were 71% ± 2%, 66% ± 2%, and 66% ± 2% for CP, AutoKB, and HybridKB (both p < 0.01). For larger patients, the corresponding values were 75% ± 3%, 69% ± 2%, and 68% ± 2% (both p < 0.01). D2% of the planning target volume increased in AutoKB, reaching 122% ± 2% (p < 0.001) vs. 117% ± 3% in CP for standard configurations, and 126% ± 2% (p < 0.001) vs. 117% ± 3% in CP for arms configurations. HybridKB was on par with CPs.

**Conclusions:**

A single KB model enabled effective planning for multi-isocenter TMLI, including anatomies requiring separate isocenters for the arms. Fully automated KB provided suboptimal dose distributions. KB with manual refinements reduced planner dependence and improved plan quality.

## Highlights

• A knowledge-based (KB) model was developed for multi-isocenter planning of TMLI.
• KB planning reduced mean doses to organs at risk and improved workflow efficiency.
• Manual refinements enhanced KB plans, addressing hotspots and plan quality.
• KB enabled effective planning for patient cohorts with different anatomies.

## 1 Introduction

Total marrow and lymphoid irradiation (TMLI) is an advanced radiotherapy (RT) technique used as part of conditioning regimen for patients undergoing hematopoietic cell transplantation in acute leukemia [1]. Radiation delivery serves as an effective means of immunosuppression to prevent rejection of donor cells and reduce transplant-related mortality [2,3]. Unlike Total body irradiation (TBI), which exposes the entire body to radiation, TMLI specifically targets the bone marrow, lymph node chains and spleen. This tissue-sparing approach offers a promising alternative to TBI for reducing the risk of severe toxicities to organs-at-risk (OARs) while maintaining therapeutic efficacy [[4], [5], [6]].

TMLI delivery is made possible through image-guided intensity-modulated radiotherapy (IMRT). Early TMLI attempts involved helical tomotherapy (HT) [7] and static IMRT fields using standard linacs [8,9], proving that doses to OARs were reduced compared to TBI. A substantial technological innovation emerged with volumetric modulated arc therapy (VMAT), achieving equivalent efficacy to HT and IMRT in terms of OAR sparing [[10], [11], [12]]. In addition, the main advantage of VMAT is its compatibility with most modern linacs, potentially enabling a widespread adoption of TMLI [1].

However, TMLI is currently limited by several drawbacks related to extensive clinical experience and time required for treatment planning. Specifically, for each patient, the planner selects a multi-isocenter configuration based on his personal experience and local protocols. Then, the plan is optimized via inverse planning in which the planner can adapt the objective weights to maximize the dose to the target while reducing the dose to OARs.

Knowledge-based (KB) planning models are emerging as promising tools to streamline and improve complex treatment planning processes. These models leverage clinically approved plans to build and train mathematical algorithms capable of predicting dose-volume histogram (DVH) objectives for OARs and priority for both OARs and targets in new patients. KB planning has demonstrated its effectiveness in optimizing the planning process for various anatomical sites, including the prostate [[13], [14], [15]], head and neck [[16], [17], [18], [19]], and lungs [20], although regular updates are necessary to ensure their clinical usability over time [21]. Notably, Ahn et al. developed a KB model for total marrow irradiation (TMI), addressing challenges related to the use of multiple isocenters [22]. However, TMLI and TMI pose additional challenges for KB planning, as the number and positioning of isocenters are tailored to the patient’s anatomy [23,24]. In particular, for patients with large body size in lateral direction, two specific isocenters are placed on the arms, therefore producing two distinct patient groups.

This study aims to develop and validate a KB model for VMAT-TMLI, with two primary objectives: determining whether fully automated KB planning can achieve clinically acceptable dose distribution or requires manual refinement, and assessing the model versatility across different patient anatomies.

## 2 Materials and methods

### 2.1 Patient selection

Fifty-one consecutive patients treated with TMLI between 2020 and 2023 were considered to ensure a homogeneous study cohort, representing our most recent clinical practice in TMLI. In all cases, the clinical target volume (CTV) for lymph nodes was consistently delineated according to established internal guidelines [25], with plans demonstrating improved dosimetric quality and reduced complexity, compared to historical plans [24].

**Fig. 1:**
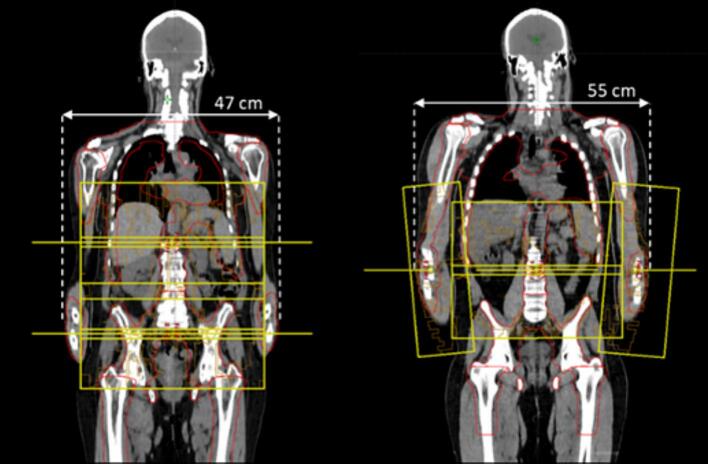
Coronal view of a patient without isocenters on the arms, i.e., body configuration (left), and a patient with two additional isocenters on the arms, i.e., arms configuration (right).

Two patient categories were defined according to the treatment field geometry and isocenters positioning used, as shown in Fig. 1. The first group was treated with 5 isocenters along the body (body configuration), while the second with 4 isocenters along the body and 2 along the arms (arms configuration). The arms configuration was used when the width of thorax and arms exceeded 47 cm, i.e., much larger than the maximum field aperture of 40 cm and preventing optimal target coverage [26].

Of the selected 51 patients, 30 (10 women, 20 men) treated using the body configuration were used to train a KB model. The remaining 21 patients formed two validation cohorts including, respectively, 10 patients treated with the body configuration (body validation) and 11 patients treated with the arms configuration (arms validation). The limited number of cases involving the arms configuration precluded the creation of adequately sized and balanced training and validation subsets for this setup. Thus, model training was restricted to the body configuration, which accounted for the majority of treatments.

### 2.2 Total marrow and lymphoid irradiation planning

TMLI patients were treated in accordance with the guidelines approved by the Institutional Ethics Committee of IRCCS Humanitas Research Hospital (ID 2928, 26th Jan 2021; ClinicalTrials.gov Identifier: NCT04976205) [27]. The CTV included the skeletal bones (including the chest wall), the spleen, and all lymph node chains. The mandible and hands were excluded from the CTV. The planning target volume (PTV) was obtained though isotropic expansion of the CTV of bone marrow by 2 mm (plus 8 mm for the arms and legs), and the CTV of spleen and lymph nodes by 5 mm. We refer to our protocol for additional details [25].

Plans were optimized for a Varian TrueBeam equipped with a Millennium MLC with leaf width of 5 mm at the isocenter in the inner 20 cm, and 10 mm for the outer 2 × 10 cm (i.e., a total of 40 cm). For all VMAT arcs along the body, the collimator angle was set to 90°, i.e., perpendicular to the cranial-caudal direction. Adjacent arcs overlapped by at least 2 cm on either side to minimize discrepancies between the delivered and planned doses caused by patient misalignments [28]. Plan optimizations were conducted with the Photon Optimizer (PO) V15 implemented in the Eclipse Treatment Planning System (TPS) [24]. Dose distributions were computed with the Analytical Anisotropic Algorithm (AAA) V15, with a calculation grid resolution of 2.5 mm. All plans had a dose prescription of 2 Gy in single fraction and were normalized such that 98 % of the PTV received 98 % of the prescribed dose (PTV 98 % = 98 %).

### 2.3 Model creation

The KB model was created using RapidPlan V16. All the target and non-target structures were included in the model and, for each structure, the optimization objectives and priorities were selected. The complete list and information are reported in Table S1 of the Supporting information.

Regarding the target, 10 structures were added: the total PTV, 5 sub-structures of PTV for specific body regions (PTV Brain, PTV Thorax, PTV Arms, PTV Abdomen, and PTV Legs), and 4 junction structures (PTV J100%, PTV J75%, PTV J50%, and PTV J25%) used for the planning of the lower extremities [29]. According to a study conducted at our institute [29], defining 4 structures where the dose decreases from 100 % to 25 % of the prescribed dose is a robust way to create a dose gradient and produce a suitable plan sum.

Regarding the non-target regions, in addition to the body structure, 21 other structures were included: bladder, body free (created from the body with a 3 mm inner margin and cropped with the PTV using a margin of 35 mm), bowel, brain, eyes, heart, HT1 (“healthy tissue” ring created using the PTV with an outer margin of 15 mm and inner margin of 3 mm), HT2 (created using the PTV with an outer margin of 30 mm and inner margin of 17 mm), kidney left, kidney right, larynx, lens, liver, lung left, lung right, oral cavity, parotids, rectum, stomach, testis and thyroid. For small bilateral structures, i.e. eyes, lens and parotids, both structures were matched to the same model structure, while for larger bilateral structures, i.e. kidneys and lungs, two model structures were defined for the right and the left organ. Eighteen out of 620 OAR contours were excluded from the training data due to being either missing (13 cases) or not fully compliant with institutional contouring guidelines, as assessed through qualitative evaluation by a radiation oncologist.

All the target structures, excluding junction targets, were related to an upper objective with 110 % dose at a volume of 0 %, and a lower objective with 99 % dose at a volume of 100 %, both with a fixed priority of 120 (target priorities range 50–200). In order to achieve a gradual decrease in the junction region, appropriate lower and upper objectives of PTV J100%, PTV J75%, PTV J50%, and PTV J25% were defined. For all non-target structures, except for body and body free, a mean, an upper gEUD and a line objective were included with generated volume, dose and priority.

### 2.4 Model validation

To validate the model, the clinical plans (CPs) were compared with the KB plans, normalizing both to PTV 98 % = 98 %. KB plans were optimized with PO V16 and all dose distributions were computed using AAA V16.

For each patient, two planning approaches were explored: (i) a fully automated strategy without manual adjustments (AutoKB), and (ii) a hybrid approach combining KB automation with manual refinement during the inverse optimization (HybridKB). Notably, manual refinement was performed by a planner with no prior experience in TMLI under the supervision of an experienced medical physicist, whereas CPs were optimized by a planner with expertise in TMLI. This design enabled an assessment of the HybridKB approach’s capacity to produce high-quality plans regardless of the planner’s prior experience with TMLI.

During the AutoKB optimization, DVH estimations and automatic objectives were generated based on the model without any modification. Following normalization of the AutoKB plan, ensuring the average dose to the target equaled 100 % of the prescribed dose, new structures were delineated to improve PTV coverage and mitigate dosimetric hotspots (see Table 1). These additional structures were included into the HybridKB plans, which were further refined by adjusting the model-generated parameters to achieve the desired dose distribution.

**Table 1:** Additional structures with the relative objectives and priorities used to increase PTV coverage and reduce dosimetric hotspots.

**Structure**	**Description**	**Objective**	**Volume [%]**	**Dose [%]**	**Priority**	**gEUD a**
Dose 95 %	PTV volume where the delivered dose is not covered by the isodoses of 95 %.	Lower	100 %	99 %	96	−
Dose 100 %	Volume outside the PTV + 5 mm where the delivered dose ≥ 100 % of the prescribed dose.	Upper gEUD	−	95	96	40
Dose 105 %	Volume where the delivered dose is ≥ 105 %.	Upper	0 %	110 %	96	−

### 2.5 Statistics

The model quality was evaluated by checking the model goodness of fit statistics for each structure, using the coefficient of determination $R^{2}$ and the chi square $\chi^{2}$. Dosimetric parameters, such as D2%, V95% and mean dose for the PTV and major critical structures (bladder, body, bowel, heart, kidneys, liver and lungs) were analyzed.

Paired t-tests were conducted to identify differences in dosimetric parameters, after ensuring that data followed a normal distribution using the Shapiro-Wilk test. To comprehensively evaluate dose distribution in healthy tissue, the overall mean dose for major OARs was calculated for each validation set as a weighted average of the mean doses to individual structures, with weights inversely proportional to their standard deviation (SD). A *t*-test was applied to assess differences in the overall mean dose. The significance level was set to 0.05 throughout the whole study, with Bonferroni’s correction applied to reduce false discovery rate from multiple comparisons.

## 3 Results

Table 2 summarizes the coefficient of determination $R^{2}$ and the chi square $\chi^{2}$ together with the number of trained structures and the number of potential outliers for the most relevant structures. $R^{2}$ values varied from 0.21 (thyroid) to 0.84 (stomach), while $\chi^{2}$ values ranged from 1.05 (lens) to 1.29 (larynx). Potential outliers were identified within each structure using these statistical measures and then closely evaluated in relation to the patient’s anatomy and dose distribution. Eyes exhibited the highest number of outliers (41 %), likely due to being treated as a single structure.

**Table 2:** Summary of the model training. The coefficient of determination $R^{2}$ ranges between 0 and 1, with higher values indicating better performances. For the chi square $\chi^{2}$, values closer to 1 indicate better performance.

**Structure**	**Coeff. of Determination**	**Chi Square**	**Matched structures**	**Outliers**
Bladder	0.32	1.1	30	2
Bowel	0.60	1.2	30	0
Brain	0.37	1.1	30	2
Eyes	0.57	1.1	58	24
Heart	0.35	1.1	30	4
Kydney_L	0.66	1.1	30	7
Kydney_R	0.73	1.1	30	9
Larynx	0.73	1.3	25	6
Lens	0.24	1.0	55	7
Liver	0.65	1.2	30	3
Lung_L	0.50	1.0	30	4
Lung_R	0.48	1.1	30	9
Oral cavity	0.33	1.1	29	7
Parotids	0.67	1.1	56	5
Rectum	0.50	1.1	30	8
Stomach	0.84	1.1	30	5
Testis	0.42	1.1	20	5
Thyroid	0.20	1.0	29	5

**Fig. 2:**
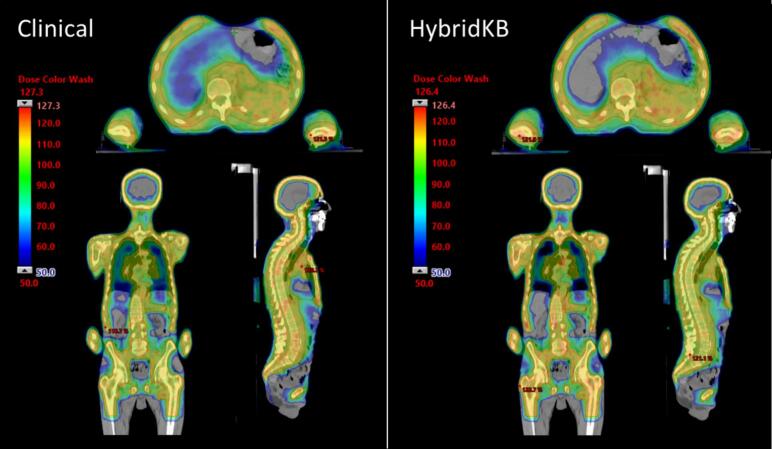
An example of CP (left) and HybridKB performance (right) in axial, coronal, and sagittal planes. Dose color wash ranges from 50% to maximum dose.

Regarding the validation against patients treated with body configuration (Table 3), the PTV D2% was 117 %±3%, 122 %±2%, and 119 %±2% for, respectively, CP, AutoKB, and HybridKB. Mean doses to major OARs were 71 %±2%, 66 %±2%, and 66 %±2% for, respectively, CP, AutoKB, and HybridKB (both p < 0.01). Significant differences between AutoKB and HybridKB indicate that manual refinement was necessary for reducing hotspots in the target and marginally improving dosimetric results for OARs. Notably, no significant differences were observed between CP and HybridKB. Fig. 2 shows a comparison between CP and HybridKB for a representative case.

**Table 3:** Dosimetric results as mean ± SD for the body validation. ^(1)^ p-value is between CP and HybridKB. ^(2)^ p-value is between AutoKB and HybridKB.

**Structure**	**Parameter**	**CP [%] ^(1)^**	**AutoKB [%] ^(2)^**	**HybridKB [%]**
PTV	Mean	107 ± 2 *p = 0.006*	111 ± 1 ***p < 0.001***	109 ± 1
	D2%	117 ± 3 *p = 0.031*	122 ± 2 ***p < 0.001***	119 ± 2
	V95%	99.5 ± 0.2 *p = 0.003*	99.2 ± 0.2 ***p < 0.001***	99.3 ± 0.2
Bladder	Mean	60 ± 7 *p = 0.021*	55 ± 6 *p = 0.581*	56 ± 7
Body	Mean	68 ± 4 *p = 0.029*	69 ± 3 ***p < 0.001***	67 ± 3
Bowel	Mean	71 ± 5 *p = 0.116*	69 ± 5 ***p < 0.001***	68 ± 4
Heart	Mean	65 ± 7 *p = 0.040*	62 ± 7 *p = 0.732*	62 ± 7
Kidneys	Mean	52 ± 10 *p = 0.016*	50 ± 7 *p = 0.481*	50 ± 7
Liver	Mean	73 ± 4 *p = 0.010*	68 ± 4 *p = 0.181*	68 ± 4
Lungs	Mean	82 ± 4 *p = 0.326*	82 ± 7 ***p < 0.001***	81 ± 6

Regarding the validation against patients treated with arms configuration (Table 4), the PTV D2% was 117 %±3%, 126 %±2%, and 119 %±1% for, respectively, CP, AutoKB, and HybridKB. Mean doses to major OARs were 75 %±3%, 69 %±2%, and 68 %±2% for, respectively, CP, AutoKB, and HybridKB (both p < 0.01). As in the body validation, the AutoKB approach preserved OARs, but increased dosimetric hotspots in the PTV. This resulted in overall hotter AutoKB plans, as highlighted by the significant increase in mean dose to the Body. No significant differences were found between CP and HybridKB, except for a decrease in mean dose to the bladder.

**Table 4:** Dosimetric results as mean ± SD for the arms validation. ^(1)^ p-value is between CP and HybridKB. ^(2)^ p-value is between AutoKB and HybridKB.

**Structure**	**Parameter**	**CP [%] ^(1)^**	**AutoKB [%] ^(2)^**	**HybridKB [%]**
PTV	Mean	108 ± 2 *p = 0.056*	113 ± 1 ***p < 0.001***	109 ± 1
	D2%	117 ± 3 *p = 0.032*	126 ± 2 ***p < 0.001***	119 ± 1
	V95%	99.3 ± 0.2 *p = 0.006*	99.2 ± 0.1 ***p < 0.001***	99.4 ± 0.1
Bladder	Mean	67 ± 8 ***p = 0.002***	60 ± 6 *p = 0.049*	59 ± 6
Body	Mean	68 ± 6 *p = 0.079*	69 ± 4 ***p < 0.001***	66 ± 4
Bowel	Mean	70 ± 5 *p = 0.068*	70 ± 5 ***p < 0.001***	67 ± 5
Heart	Mean	73 ± 9 *p = 0.017*	64 ± 7 *p = 0.171*	65 ± 7
Kidneys	Mean	58 ± 12 *p = 0.003*	52 ± 12 *p = 0.308*	51 ± 12
Liver	Mean	76 ± 7 *p = 0.008*	68 ± 5 *p = 0.036*	68 ± 5
Lungs	Mean	87 ± 6 *p = 0.004*	85 ± 6 *p = 0.006*	84 ± 6

## 4 Discussion

This study aimed to evaluate the performance of a KB model for TMLI to assess whether fully automated optimizations yield clinically acceptable results or require manual refinements. Furthermore, it investigated the feasibility of using a single model for different geometric setups, i.e., with or without isocenters on arms. KB planning combined with manual refinement allowed to significantly reduce the mean dose to major OARs for the body configuration (71 %±2% vs. 66 %±2%, p < 0.01), while maintaining target coverage. Moreover, the model proved effective also for the arms configuration (mean dose to major OARs 75 %±3% vs. 68 %±2%, p < 0.01), with suboptimal target coverage only in a few instances. Notably, KB planning reduced planner dependence, enabling to achieve a dosimetric quality comparable to that of CPs optimized by an expert in TMLI.

Typically, KB models require a large number of training cases to manage variability in patient anatomy, as well as in the shape, size, and location of the target. Notably, KB models for head and neck have been developed using data sets of more than 100 patients [18,19]. However, Caricato et al. has demonstrated that reducing the sample size to 60 cases can still preserve the quality and predictive capability of KB models for craniospinal irradiation, even in multicentric settings [30]. In addition, Ahn et al. reported that there are moderate differences in interpatient variation to support relatively small datasets for TMLI [22]. Unlike general tumors, which exhibit wide variability, the PTV defined by bone structures presents relatively consistent target-OAR geometry. On the other hand, PTV definition in TMLI may still vary considerably due to the lack of global consensus on contouring, particularly for the CTV of the lymph node chains (CTV LN). At our institute, since March 2022, the CTV LN has been delineated according to internal guidelines [25]. Given that homogeneity in target definition is crucial to reduce inter-patient variability, we decided to select the most recently treated patients to train our KB model. Additionally, these patients were characterized by a reduced plan complexity and higher dosimetric quality compared to the earlier ones [24].

The model validation, as well as the extraction of data during model configuration was extremely time-consuming due to the large number of structures and their extent. Indeed, TMLI involves more structures than conventional RT treatments. For instance, the KB model for head and neck developed by Fogliata et al. [17] included 3 target structures and 12 OARs, while our KB model encompassed 10 targets and 18 OARs. The coefficient of determination $R^{2}$ for the thyroid was notably low at 0.21, likely due to its frequent overlap with the target. The eyes had the highest number of outliers, with 24 out of 58 matched structures, potentially because they were treated as a single model structure despite being two distinct volumes. Thus, the large number of outliers rendered the model predictions for the eyes unreliable, and optimization objectives were set manually during the planning process. Interestingly, the lenses had fewer outliers compared to the eyes, suggesting that dose-volume constraints generated by the model for the lenses could serve as guidelines for defining constraints on the eyes.

The number of outliers in a KB model for TMLI is a crucial factor, as highlighted by Ahn et al [22]. In their study, TMLI delivery was achieved using three isocenters to cover sub-target volumes. Separate plans were created and optimized for each isocenter, and these were subsequently merged to obtain the final treatment plan. This multi-plan strategy breaks down the complex planning of TMLI into manageable sub-problems. However, it poses several challenges, particularly due to overlapping fields from different plans, which can result in suboptimal doses and a large number of outliers in the junction regions (e.g., kidneys and bowel). To address these limitations, the authors proposed using Eclipse V16 for KB model development, which supports both the optimization and dose estimation for multiple isocenters within a single plan. In our study, we employed RapidPlan V16 to simultaneously optimize 5–6 isocenters in a unique plan, resulting in a limited number of outliers for critical structures, particularly the kidneys and bowel. Moreover, the chi square values (see Table 2) indicate good homogeneity within the training set. Although this may be influenced by the limited sample size, potentially affecting generalizability, these values align with those reported in the literature [17,[20], [21], [22],^30^].

To evaluate the performance of the KB model for patients with the arms configuration, a validation using a cohort of patients treated with this setup was conducted. The model achieved precise dose distributions for the target in most cases and, in a few instances, the target coverage was suboptimal even with HybridKB, especially on the chest and arms. Despite these challenges, the model demonstrated its suitability for patients in this cohort. In particular, the predictive capability of the model for OARs within the field of view of the additional arcs was not compromised. Although the ideal approach would involve developing a KB model specifically tailored for the arms configuration, this is currently unfeasible due to the insufficient number of patients treated with this setup reflecting our most recent clinical practice. Given the increasing number of TMLI patients, it is expected that sufficient data will soon become available. However, the increased workload associated with model development and maintenance should be taken into account.

The dose distribution of AutoKB plans was suboptimal due to increased dosimetric hotspots. In addition, KB objectives often pushed the optimization excessively to reduce the dose to the OARs, compromising the target coverage. On the other hand, HybridKB plans were characterized by improved OAR sparing compared to CPs, accurate target coverage, and fewer dosimetric hotspots than AutoKB, resulting in an overall improvement in dose statistics. Thus, the combination of KB with manual refinement proved essential for optimizing TMLI planning, underscoring the value of human oversight in leveraging KB approaches.

The main limitation of this study was the restricted number of patients available for model training and validation. Additionally, the model was not validated using data from an external center, which would be crucial to assess potential biases related to specific characteristics of varying clinical settings. Furthermore, CPs were optimized using Eclipse V15, whereas the KB model was developed with Eclipse V16, possibly favoring KB plans in the comparison due to the expected performance improvements associated with the newer software version. In an effort to enhance model performance, it could be beneficial to re-optimize all plans in the training data set with this KB model and subsequently train a new KB model using these plans. This iterative approach could maximize the limited number of TMLI patients available and improve model quality [30,31]. An alternative solution could be increasing the library size by re-optimizing older plans. However, this approach requires aligning the target and OAR contours with current clinical practices, which would increase the workload compared to re-optimization alone.

In conclusion, a single KB model was developed to optimize multi-isocenter TMLI plans across diverse patient anatomies. The fully automated approach significantly reduced mean doses to healthy tissues but compromised target coverage. The combination of KB and manual refinement produced plans of comparable quality to CPs. The KB model provided reference dose distributions, reducing dependence on planner expertise even for a highly complex treatment such as TMLI.

## Declaration of competing interest

The authors declare that they have no known competing financial interests or personal relationships that could have appeared to influence the work reported in this paper.

Pietro Mancosu is an Editorial Board Member for Physics and Imaging in Radiation Oncology and was not involved in the editorial review or the decision to publish this article.
