# Supplementary material for: Knowledge-based model for automated multi-isocenter total marrow and lymphoid irradiation planning across standard and large patient anatomies
Source: Phys Imaging Radiat Oncol. 2025 May 20;34:100781. doi: 10.1016/j.phro.2025.100781 (PMC12151680; doi:10.1016/j.phro.2025.100781)
Supplement: Supplementary Data 1 [file mmc1.docx]

### **Supporting Information**

The following section lists all the structures included in the model, along with their respective objectives and priorities.

| **Structure** | **Objective** | **Volume [%]** | **Dose [%]** | **Priority** | **gEUD a** |
| --- | --- | --- | --- | --- | --- |
| PTV_Abdomen | Upper | 0% | 110% | 120 |  |
|  | Lower | 100% | 99% | 120 |  |
| PTV_Arms | Upper | 0% | 110% | 120 |  |
|  | Lower | 100% | 99% | 120 |  |
| PTV_Brain | Upper | 0% | 110% | 120 |  |
|  | Lower | 100% | 99% | 120 |  |
| PTV_J100% | Upper | 0% | 110% | 120 |  |
|  | Lower | 100% | 99% | 120 |  |
| PTV_J25% | Upper | 0% | 110% | 120 |  |
|  | Lower | 100% | 99% | 120 |  |
| PTV_J50% | Upper | 0% | 110% | 120 |  |
|  | Lower | 100% | 99% | 120 |  |
| PTV_J75% | Upper | 0% | 110% | 120 |  |
|  | Lower | 100% | 99% | 120 |  |
| PTV_Thorax | Upper | 0% | 110% | 120 |  |
|  | Lower | 100% | 99% | 120 |  |
| UpperLegsPTVNoJ | Upper | 0% | 110% | 120 |  |
|  | Lower | 100% | 99% | 120 |  |
| UpperPTVNoJ | Upper | 0% | 110% | 120 |  |
|  | Lower | 100% | 99% | 120 |  |
| Bladder | Mean |  | Generated | Generated |  |
|  | Upper gEUD |  | Generated | Generated | 40 |
|  | Line | Generated | Generated | Generated |  |
| Body | Upper gEUD |  | Generated | Generated | 40 |
|  | Line | Generated | Generated | Generated |  |
| Body_free | Upper gEUD |  | Generated | Generated | 40 |
|  | Line | Generated | Generated | Generated |  |
| Bowel | Mean |  | Generated | Generated |  |
|  | Upper gEUD |  | Generated | Generated | 40 |
|  | Line | Generated | Generated | Generated |  |
| Brain | Mean |  | Generated | Generated |  |
|  | Upper gEUD |  | Generated | Generated | 40 |
|  | Line | Generated | Generated | Generated |  |
| Eyes | Mean |  | Generated | Generated |  |
|  | Upper gEUD |  | Generated | Generated | 40 |
|  | Line | Generated | Generated | Generated |  |
| Heart | Mean |  | Generated | Generated |  |
|  | Upper gEUD |  | Generated | Generated | 40 |
|  | Line | Generated | Generated | Generated |  |
| HT1 | Mean |  | Generated | Generated |  |
|  | Upper gEUD |  | Generated | Generated | 40 |
|  | Line | Generated | Generated | Generated |  |
| HT2 | Mean |  | Generated | Generated |  |
|  | Upper gEUD |  | Generated | Generated | 40 |
|  | Line | Generated | Generated | Generated |  |
| Kydney_L | Mean |  | Generated | Generated |  |
|  | Upper gEUD |  | Generated | Generated | 40 |
|  | Line | Generated | Generated | Generated |  |
| Kydney_R | Mean |  | Generated | Generated |  |
|  | Upper gEUD |  | Generated | Generated | 40 |
|  | Line | Generated | Generated | Generated |  |
| Larynx | Mean |  | Generated | Generated |  |
|  | Upper gEUD |  | Generated | Generated | 40 |
|  | Line | Generated | Generated | Generated |  |
| Lens | Mean |  | Generated | Generated |  |
|  | Upper gEUD |  | Generated | Generated | 40 |
|  | Line | Generated | Generated | Generated |  |
| Liver | Mean |  | Generated | Generated |  |
|  | Upper gEUD |  | Generated | Generated | 40 |
|  | Line | Generated | Generated | Generated |  |
| Lung_L | Mean |  | Generated | Generated |  |
|  | Upper gEUD |  | Generated | Generated | 40 |
|  | Line | Generated | Generated | Generated |  |
| Lung_R | Mean |  | Generated | Generated |  |
|  | Upper gEUD |  | Generated | Generated | 40 |
|  | Line | Generated | Generated | Generated |  |
| OralCavity | Mean |  | Generated | Generated |  |
|  | Upper gEUD |  | Generated | Generated | 40 |
|  | Line | Generated | Generated | Generated |  |
| Parotids | Mean |  | Generated | Generated |  |
|  | Upper gEUD |  | Generated | Generated | 40 |
|  | Line | Generated | Generated | Generated |  |
| Rectum | Mean |  | Generated | Generated |  |
|  | Upper gEUD |  | Generated | Generated | 40 |
|  | Line | Generated | Generated | Generated |  |
| Stomach | Mean |  | Generated | Generated |  |
|  | Upper gEUD |  | Generated | Generated | 40 |
|  | Line | Generated | Generated | Generated |  |
| Testis | Mean |  | Generated | Generated |  |
|  | Upper gEUD |  | Generated | Generated | 40 |
|  | Line | Generated | Generated | Generated |  |
| Thyroid | Mean |  | Generated | Generated |  |
|  | Upper gEUD |  | Generated | Generated | 40 |
|  | Line | Generated | Generated | Generated |  |

**Table S1** Objectives and priorities in the model.
